# Bam complex-mediated assembly of bacterial outer membrane proteins synthesized in an in vitro translation system

**DOI:** 10.1038/s41598-020-61431-2

**Published:** 2020-03-12

**Authors:** Sunyia Hussain, Janine H. Peterson, Harris D. Bernstein

**Affiliations:** 1 Genetics and Biochemistry Branch, National Institute of Diabetes and Digestive and Kidney Diseases, National Institutes of Health, Bethesda, MD 20892-0538 USA; 2 Present Address: TetraGenetics, Inc., 91 Mystic St., Arlington, MA 02474 USA

**Keywords:** Biochemistry, Biological techniques, Microbiology

## Abstract

Bacterial outer membrane proteins (OMPs) contain a unique “β barrel” segment that is inserted into the membrane by the barrel assembly machinery (Bam) complex by an unknown mechanism. OMP assembly has been reconstituted *in vitro*, but assembly reactions have involved the use of urea-denatured protein purified from inclusion bodies. Here we show that the *E. coli* Bam complex catalyzes the efficient assembly of OMPs synthesized de novo in a coupled *in vitro* transcription/translation system. Interestingly, the *in vitro* translated forms of the OMPs we analyzed were assembled more rapidly and were effectively engaged by fewer periplasmic chaperones than their urea-denatured counterparts. Taken together, our results strongly suggest that the mode of production influences the conformational states sampled by OMPs and thereby affects their recognition by both chaperones and the Bam complex. Besides providing insights into OMP biogenesis, our work describes a novel, streamlined method to reconstitute OMP assembly *in vitro*.

## Introduction

Most proteins that are inserted into the outer membrane (OM) of Gram-negative bacteria are anchored to the membrane by a unique membrane spanning segment referred to as a “β barrel”. β barrels are essentially amphipathic β sheets that fold into a closed cylindrical structure held together by a network of hydrogen bonds. Unlike the α-helical membrane spanning segments that are typically found in most integral membrane proteins, β barrels presumably cannot be stably integrated into the OM before they fold and expose a hydrophobic exterior. Although they share a common design, OM proteins (OMPs) are structurally rather heterogeneous. The β barrels themselves vary in size considerably from 8–36 β strands^1,2^. While some β barrels are empty, others contain an embedded polypeptide. Furthermore, some OMPs form homooligomers or contain a soluble periplasmic or extracellular domain linked to the β barrel domain.

The key steps in the OMP assembly pathway have been well established. After OMPs are translocated across the inner membrane though the Sec machinery, they interact with a variety of molecular chaperones including Skp, SurA and DegP, a protein that also functions as a protease^3–7^. While the chaperones differ dramatically in structure and their substrate binding properties are poorly understood, it is generally believed that they maintain OMPs in an assembly-competent conformation. Insertion into the OM is then catalyzed by a heterooligomer called the barrel assembly machinery (Bam) complex that consists of a variable number of subunits^8–10^. In *E. coli* the Bam complex consists of BamA, an integral OMP that contains a β barrel domain and five periplasmic polypeptide transport-associated (POTRA) domains, and four lipoproteins (BamB-E) that bind to the POTRA domains^9,11,12^.

Although the structure of the Bam complex was recently solved^13–16^, the mechanism by which it catalyzes the membrane insertion of OMPs is unknown. All of the current models center on striking evidence that an unstable connection between the first and last β strands of the BamA β barrel enables it to open laterally^17,18^. In the “threading” or “budding” model, it has been proposed that OMPs enter the pore of the BamA β barrel in an unfolded conformation and then insert into the lipid bilayer in a stepwise fashion through the lateral opening. Recent results suggest that at least some OMPs undergo significant folding inside the BamA β barrel before they are released into the membrane^19^. An alternative model (“assisted” model) postulates that the opening of the BamA β barrel facilitates the membrane integration of folded or partially folded client proteins simply by perturbing the lipid bilayer. While both models are supported by various lines of experimental evidence, a recent analysis of a stalled OMP assembly intermediate led to a third model (“swing” model) in which the BamA β barrel opens and forms an asymmetric hybrid barrel with partially folded client proteins. In this model a stable interface between the first β strand of BamA and the last β strand of the client holds the two barrels together while the N-terminus of the client moves along the C-terminal strands of BamA into the OM^20^.

OMP assembly has not only been analyzed *in vivo*, but has also been investigated *in vitro* using purified components. Multiple studies conducted over the last 25 years have reported the spontaneous assembly of a variety of urea-denatured *E. coli* OMPs into pure lipid vesicles^21–24^. In general, however, assembly requires the use of non-physiological conditions (e.g., high pH) and time frames (hours to days). Furthermore, assembly is very sensitive to the surface charge, fluidity and thickness of the lipid bilayers and is often incompatible with abundant native lipids such as phosphatidylethanolamine (PE)^23,25–27^. More recent studies have shown that when the Bam complex is purified and reconstituted into proteoliposomes it catalyzes the efficient assembly of several different urea-denatured OMPs into the vesicles within minutes around neutral pH in the presence of SurA^28–30^. Interestingly, neither the efficiency nor the kinetics of assembly is significantly affected by the lipid composition of the proteoliposomes^30^.

Although the development of a Bam complex-dependent assay provides an important tool for studying the mechanism by which OMPs are assembled *in vivo*, a possible drawback of the current method is that OMPs are added to the reaction as artificially denatured full-length polypeptides. In living cells, OMPs are translocated into the periplasm in an N-to-C terminal fashion that may affect their conformation. Here we show that OMPs synthesized de novo in a coupled *in vitro* transcription/translation system that simulates this directionality can also be assembled efficiently by the Bam complex. Interestingly, several results that emerged from our experiments raised the intriguing possibility that *in vitro* translated OMPs adopt a distinct conformation that affects their interaction with chaperones and enhances their recognition by the Bam complex. From a practical perspective, our work also demonstrates that an *in vitro* translation-based approach can be used to bypass the labor-intensive expression and purification of OMPs *in vivo* and to simplify the analysis of OMP assembly considerably.

## Results and Discussion

We used a well-established coupled transcription/translation system (the “PURE system”) to determine if the Bam complex can catalyze the assembly of de novo synthesized OMPs into proteoliposomes. The PURE system contains T7 polymerase to generate mRNA transcripts from the T7 promoter, purified *E. coli* ribosomes, and recombinant forms of all of the factors required to drive protein synthesis *in vitro*^31^. We added a plasmid that encodes one of five different *E. coli* OMPs without a signal peptide under the control of the T7 promoter to the PURE system and performed reactions at 37 °C for 30 min. Lysine residues tagged with the fluorescent dye BODIPY-FL were incorporated into the proteins during translation to facilitate their detection. SurA was added to all reactions, and OMP assembly was examined both in the presence and absence of proteoliposomes composed of the purified Bam complex and the model lipid POPC (Bam POPC proteoliposomes). We chose POPC because it produces bilayers that approximate the fluidity and hydrophobic width of the *E. coli* OM and because urea-denatured OMPs have been shown to assemble efficiently into Bam POPC proteoliposomes^30^. We were able to monitor the assembly of an “autotransporter” derivative called EspPΔ5 by assessing the autoproteolytic release of a 46 residue polypeptide that traverses its β barrel domain because cleavage occurs only after the protein is fully folded^32,33^. Otherwise we monitored assembly by exploiting the observation that in the absence of heat, fully folded OMPs generally resist SDS-denaturation and migrate more rapidly (or occasionally more slowly) than their predicted molecular weight on SDS-PAGE. We also assessed the insertion of OMPs into the proteoliposomes by testing their resistance to proteinase K (PK) digestion.

**Figure 1:**
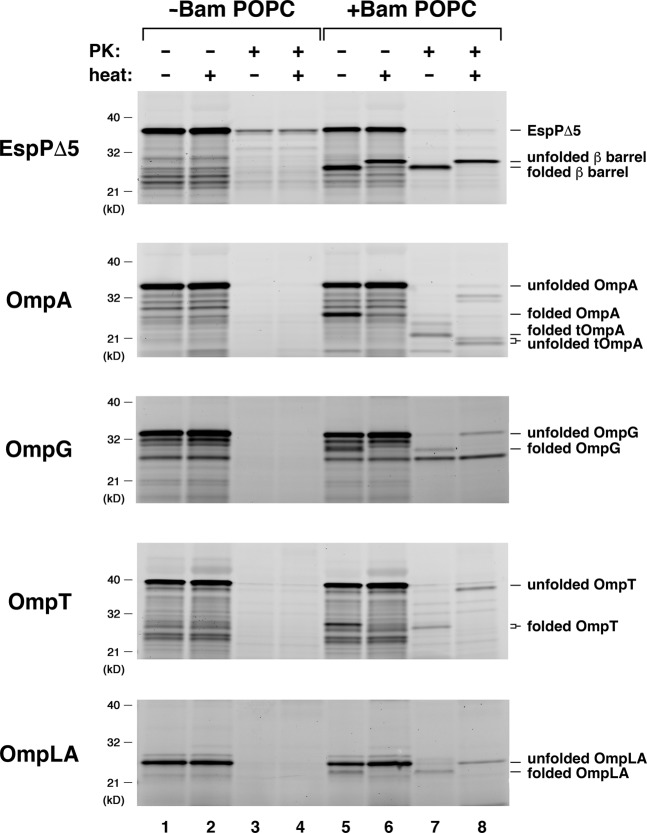
OMPs synthesized *in vitro* are assembled by the Bam complex. PURExpress coupled transcription/translation reactions supplemented with BODIPY-FL-ε-Lys-tRNA^Lys^ and SurA were programmed with a plasmid encoding the indicated OMP under the control of a T7 promoter and incubated either with or without Bam POPC proteoliposomes at 37 °C for 30 min. Aliquots were then removed from each reaction and treated with PK and/or heated to 95 °C, and proteins were resolved by SDS-PAGE. The OMPs were detected based on the incorporation of fluorescently-tagged lysine residues during synthesis.

We observed Bam complex-mediated assembly of all of the de novo synthesized OMPs we tested. Roughly half of the EspPΔ5 that was synthesized in the *in vitro* reaction underwent autocatalytic processing when Bam POPC proteoliposomes were present (Fig. 1, top gel, lane 6). The rapid migration of the cleaved β barrel in the absence of heat and its resistance to PK digestion confirmed that the protein was folded and properly inserted into the proteoliposomes (Fig. 1, top gel, lanes 5–8). A similar fraction of the urea-denatured form of the protein has been observed to assemble^30^. Based on its mobility on SDS-PAGE in the absence of heat, a slightly lower fraction of OmpA folded (Fig. 1, second gel, lane 5). As expected, PK digestion removed a large periplasmic fragment from OmpA, but a segment that likely corresponds to the β barrel domain (tOmpA) was resistant to PK digestion (Fig. 1, second gel, lanes 7–8). Significant fractions of OmpG and OmpT were also assembled (Fig. 1, third and fourth gels). Although only a small amount of folded OmpLA was observed (Fig. 1, bottom gel), the results are notable because we have not been able to detect any assembly of the urea-denatured form of this protein into Bam POPC proteoliposomes (data not shown). None of the proteins folded or attained a PK-resistant state in the absence of Bam POPC (Fig. 1, all gels, lanes 1–4). The finding that EspPΔ5 did not fold in the presence of empty POPC liposomes confirmed that assembly was mediated by the Bam complex (Fig. S1, top gel). A small amount of OmpA folded into pure POPC vesicles (much less than folded into Bam POPC proteoliposomes), but similar results were obtained when the urea-denatured form of the protein was analyzed^30^.

**Figure 2:**
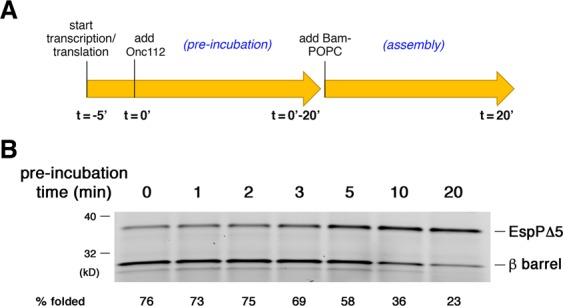
Analysis of assembly-competence of EspPΔ5 molecules synthesized *in vitro*. (**A**) Summary of the experimental strategy used to determine the duration of assembly-competence of EspPΔ5 molecules following the addition of the translation inhibitor Onc112. (**B**) A PURExpress reaction supplemented with BODIPY-FL-ε-Lys-tRNA^Lys^ and SurA was programmed with a plasmid encoding EspPΔ5 under the control of a T7 promoter and incubated at 37 °C for 5 min. Following the addition of Onc112, the reaction was incubated for a variable “pre-incubation” period of 0–20 min. At each time point an aliquot was removed and incubated with Bam POPC proteoliposomes for an additional 20 min. Fluorescently-labeled protein was then detected after SDS-PAGE. EspPΔ5 assembly was assessed by determining the percent of the protein that underwent self-cleavage.

We next wished to determine if the efficiency of OMP assembly is affected by the method of production. Because urea-denatured OMPs are added to assembly reactions as a single population of fully synthesized molecules while OMPs produced in the PURE system are synthesized continuously, we could not accurately compare folding efficiencies by determining the fraction of protein assembled after a 30 min incubation. Indeed the membrane integration of protein molecules produced *in vitro* can be strongly influenced by the time at which they are synthesized and their ability to remain assembly-competent. In addition, Western blot analysis indicated that the amount of each OMP synthesized in the PURE system in 30 min generally exceeded the amount of urea-denatured protein (0.2 μM) that we add to assembly reactions (data not shown). For these reasons it was necessary to evaluate the kinetics of assembly to obtain a more reliable comparison. Interestingly, we found in a previous study that denatured OMPs are assembled at significantly different rates that do not strictly correlate with the number of β strands^30^. To determine the rates at which OMPs produced in the PURE system are assembled, we needed to establish a method to follow the fate of a cohort of protein molecules synthesized during a short time window. To this end we conducted trial experiments in which we produced EspPΔ5 in a coupled transcription/translation reaction for only 5 min at 37 °C and then added a peptide called Oncocin (Onc112) to block further translation initiation (Fig. 2A). Onc112 blocks re-initiation by destabilizing the translation initiation complex, but does not affect translation elongation^34^. After adding the peptide, we next returned the reactions to 37 °C for various “pre-incubation” times (0–20 min) to allow previously initiated nascent polypeptide chains to be completed. At each time point aliquots were removed and mixed with Bam POPC proteoliposomes. Assembly of the protein was then monitored after 20 min. Efficient assembly was observed if the pre-incubation period was limited to 0–3 min, but the fraction of the protein that assembled gradually declined after longer pre-incubation periods (Fig. 2B). The results imply that the newly synthesized EspPΔ5 remains assembly-competent for a relatively short period of time. In addition, the finding that the level of EspPΔ5 did not significantly increase during the pre-incubation period confirms that the Onc112 peptide effectively inhibited new rounds of translation.

**Figure 3:**
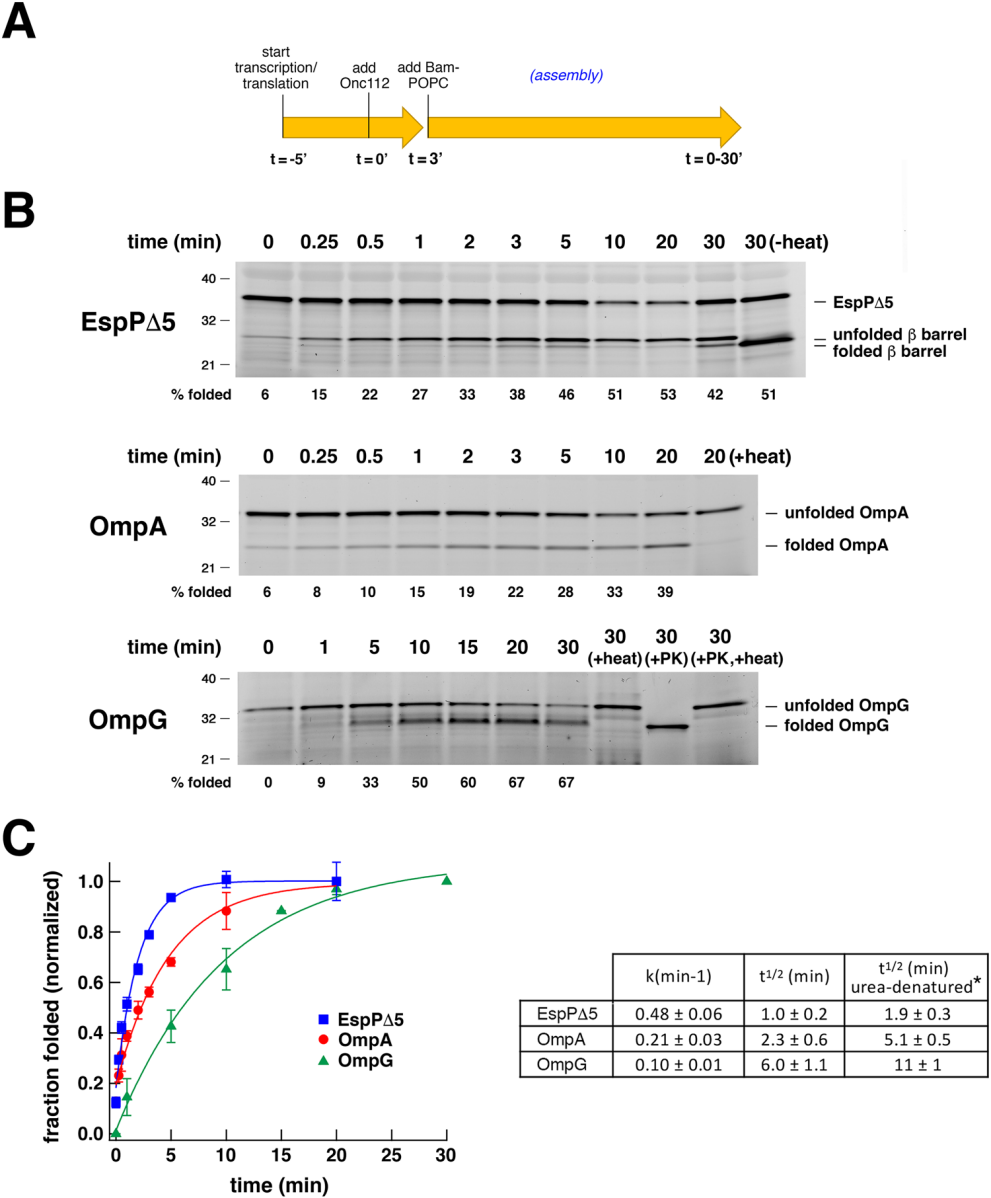
OMPs synthesized *in vitro* are assembled rapidly. (**A**) Summary of the experimental strategy used to determine the kinetics of OMP assembly. For OmpG reactions, Onc112 and Bam-POPC proteoliposomes were both added after an 8 min coupled transcription/translation reaction. (**B**) PURExpress reactions supplemented with BODIPY-FL-ε-Lys-tRNA^Lys^ and SurA were programmed with a plasmid encoding the indicated OMP under the control of a T7 promoter and incubated at 37 °C for 5 min (8 min for OmpG). Following the addition of Onc112 reactions were incubated for a further 3 min. (This step was omitted for OmpG). Bam POPC proteoliposomes were then added and aliquots were removed at various timepoints. Fluorescently-labeled protein was detected after SDS-PAGE. While EspPΔ5 assembly was assessed by determining the percent of the protein that underwent self-cleavage, the assembly of OmpA and OmpG was assessed by determining the percent of the protein that was resistant to SDS denaturation in the absence of heat. (**C**) Time course of OMP assembly based on the results of at least three experiments. The data were normalized to the maximum fraction of the protein that was assembled during each reaction (defined as 1.0). Average values (symbols) and standard deviation values (error bars) are shown. The rate constant (k) and time required to reach 50% maximum assembly (t½) for each reaction were calculated from single-exponential fits. For comparison, the t½ values for the assembly of urea-denatured forms of the same proteins that were previously calculated^30^ are shown (*).

By adapting the same basic protocol we examined the assembly kinetics of three OMPs (EspPΔ5, OmpA, and OmpG) that were synthesized in the PURE system. These three OMPs were chosen because the assembly kinetics of their urea-denatured forms was previously reported^30^. To examine EspPΔ5 and OmpA assembly, Onc112 was added to the PURE system following a 5 min incubation at 37 °C. Bam POPC proteoliposomes were added to the reactions after a further 3 min incubation, and aliquots were removed at various time points to monitor assembly (Fig. 3A). To examine the assembly of OmpG, which is more difficult to detect because it contains relatively few lysines, both Onc112 and Bam POPC proteoliposomes were added after 8 min coupled transcription/translation reactions. All of the data were normalized to the maximum fraction of each protein that was folded (defined as 100%). Remarkably, some of the EspPΔ5 and OmpA molecules were assembled immediately after the addition of the proteoliposomes, and a significant fraction of both proteins assembled within 1 min (Fig. 3B). The relative rate of assembly of the *in vitro* synthesized forms of the proteins matched the relative rate of assembly of the urea-denatured forms: EspPΔ5 was assembled the most rapidly (t½ ~ 1.0 min), OmpA was assembled at an intermediate rate (t½ ~ 2.3 min), and OmpG was assembled the most slowly (t ½ ~ 6.0 min) (Fig. 3C). Based on a comparison with the results of a previous study^30^, however, the *in vitro* translated form of each protein appeared to be assembled about two-fold more rapidly than the cognate urea-denatured form (for EspPΔ5: ~1.0 min vs. ~1.9 min; for OmpA ~2.3 min vs. 5.1 min; for OmpG 6.0 vs. 11 min). A similar disparity was observed when the assembly of *in vitro* translated and urea-denatured EspPΔ5 was examined simultaneously in a single experiment (Fig. S2A). This disparity is not due to the use of different methods to detect the two forms of the protein (Fig. S2B). Although there are fundamental differences in the components of the reactions that may account for at least part of the discrepancy, the addition of the same concentration of urea that is present in traditional OMP assembly assays to the PURE system had no effect on the kinetics of EspPΔ5 assembly (Fig. S3).

**Figure 4:**
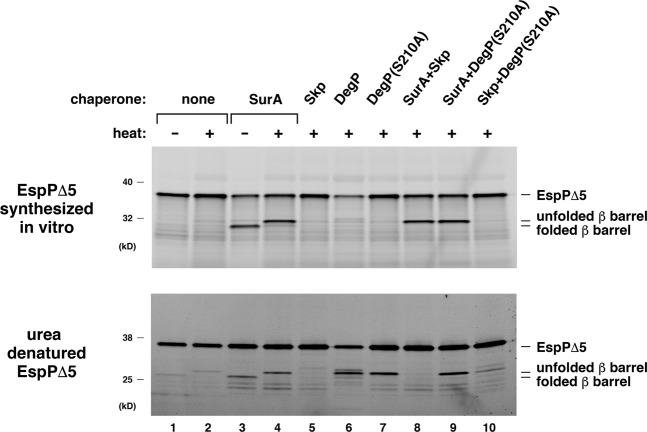
Periplasmic chaperones exert different effects on the assembly of *in vitro* synthesized and urea-denatured EspPΔ5. Top, PURExpress reactions supplemented with BODIPY-FL-ε-Lys-tRNA^Lys^ and the indicated chaperone (2 μM) or pair of chaperones (each 2 μM) were programmed with a plasmid encoding EspPΔ5 under the control of a T7 promoter and incubated with Bam POPC proteoliposomes at 30 °C for 20 min. Fluorescently-labeled protein was then detected after SDS-PAGE. Bottom, urea-denatured EspPΔ5 was added to assembly reactions that contained the indicated chaperone (2 μM) or pair of chaperones (each 2 μM) and incubated with Bam POPC proteoliposomes at 30 °C for 20 min. Proteins were then resolved by SDS-PAGE, and unprocessed EspPΔ5 and the EspPΔ5 β barrel were detected by Western blot using an antiserum generated against an EspP C-terminal peptide.

The finding that the *in vitro* translated forms of all of the OMPs we analyzed were assembled more rapidly than the cognate urea-denatured forms raised the possibility that the proteins synthesized in the PURE system adopted a distinct conformation that favors recognition by the Bam complex. We hypothesized that if the method of production significantly influences the conformation of the OMPs, then their interactions with periplasmic chaperones and the effect of the chaperones on assembly might differ. To test this idea, we examined the assembly of de novo synthesized and urea-denatured EspPΔ5 after SurA, Skp, DegP, or a DegP mutant that lacks protease but not chaperone activity^35^ [DegP (S210A)] were added to the assembly reactions. As in the experiments described above, the chaperones were added to the PURE system at the start of the coupled transcription/translation reactions. Interestingly, while only SurA promoted the assembly of the *in vitro* translated form of the protein, the assembly of the urea-denatured protein was promoted by DegP and DegP (S210) in addition to SurA (Fig. 4, lanes 1–7). As seen previously^29^, the stimulatory effect of SurA on the assembly of the urea-denatured form of the protein was completely blocked by the addition of an equimolar concentration of Skp (Fig. 4, bottom gel, lane 8). Skp also blocked assembly mediated by DegP (S210A) (Fig. 4, bottom gel, lane 10). In contrast, Skp did not affect the assembly of *in vitro* translated EspPΔ5 (Fig. 4, top gel, lane 8). Taken together, the differential effects of the chaperones that we observed strongly support our hypothesis.

The results of our study clearly demonstrate that OMPs synthesized in a well-established coupled transcription/translation system can be effectively assembled into proteoliposomes by the Bam complex. After a long incubation, the fraction of several OMPs that was assembled was comparable to that observed in previous studies in which urea-denatured forms of the proteins were analyzed. Interestingly, in an effort to determine if the method of production affects the efficiency of assembly, we obtained evidence that several OMPs were assembled significantly faster when they were synthesized *in vitro* than when they were purified from inclusion bodies and denatured. Based on the results, we propose that the *in vitro* synthesized OMPs adopt a specific conformation during or immediately after translation (perhaps through an interaction with SurA) that favors rapid assembly, while the urea-denatured proteins must sample multiple conformations before attaining a similar conformational state. The observation that SurA was the only chaperone that influenced the assembly of *in vitro* synthesized EspPΔ5 while several different chaperones stimulated or inhibited the assembly of the urea-denatured form of the protein supports the idea that the latter form is more conformationally promiscuous. Furthermore, our model is consistent with other results that suggest that OMPs begin to fold in the periplasm and only interact with the Bam complex after reaching a specific folded state^36,37^. In any case, the finding that the relative rate of assembly of EspPΔ5, OmpA, and OmpG is independent of the method of production corroborates the conclusion that assembly is strongly influenced by specific sequence and/or structure-based interactions between the Bam complex and individual client proteins.

Our results suggest that the use of the PURE system to produce OMPs to study their assembly will not only complement the traditional method of purifying OMPs from inclusion bodies, but may also offer some distinct advantages. In principle, some OMPs (e.g., OmpLA) may assemble only if they adapt specific conformations that arise during translation and that potentially mimic their conformational state inside living cells. In addition, the use of a simple plasmid-based system greatly streamlines production and bypasses the need to express proteins *in vivo* and perform labor-intensive purifications. For this reason, we expect that the PURE system will be especially valuable in medium-to-large scale experiments in which the assembly of multiple OMPs or OMP variants is analyzed. On a more sophisticated level, it should also be possible to introduce randomly mutagenized plasmids that encode OMPs into the PURE system and screen for variants that are inserted into proteoliposomes in a native state. Finally, the ability to introduce a fluorescent (or radioactive) label into a protein during its synthesis in the PURE system circumvents the need to produce an antibody to detect it by Western blot or to attach an epitope tag that might interfere with proper folding.

## Methods

### Plasmid construction, protein expression and protein purification

Plasmids in which DNA fragments that encode EspP∆5, OmpA, OmpG and OmpT without a signal peptide have been cloned into pET28b under the control of the T7 promoter have been described^29,30,38^. A plasmid that encodes OmpLA was constructed using the Gibson assembly method^39^. The OmpLA insert was generated by PCR using the primers 5′-CTTTAAGAAGGAGATATACCATGGATGCAAGAGGCAACGGTGAAAG-3′ and 5′-CGGAGCTCGAATTCGGATCCTCAAAACAAATCGTTTAGCATAAC-3′ and *E. coli* strain MC4100 genomic DNA as a template. pET28b digested with Nco I and Bam HI and a three-fold excess of the insert was then added to the Gibson assembly master mix (New England Biolabs) and incubated at 50 °C for 1 h. Plasmids pYG120 (pTRC-*bamAB*_2_*CDE*_*8His*_)^14^ and pSK257^38^ were used to express and purify the *E. coli* Bam complex and His-tagged SurA as previously described^29,30^. The Bam complex was also reconstituted into proteolipsomes containing POPC and subsequently characterized as described^30^. The gene encoding DegP with a C-terminal His tag was cloned into pET28b by Gibson assembly using the oligonucleotides 5′-CTTTAAGAAGGAGATATACCATGGCAAAAAAAACCACATTAGCACTG-3′ and 5′-CAGTGGTGGTGGTGGTGGTGCTCGAGCTGCATTAACAGGTAGATGG-3′. The S210A mutation was then introduced using the QuikChange Site-Directed Mutagenesis Kit (Stratagene). Wild-type DegP and the S210A mutant were expressed and purified by modifying a previously described protocol^40^. *E. coli* BL21(DE3) transformed with the appropriate plasmid were grown in LB containing kanamycin (50 μg/mL) overnight at 37 °C. The overnight culture was then added to 1 L fresh medium and grown to OD_600_ = 0.8. Protein expression was induced by adding 0.2 M IPTG for 3 h. Cells were then pelleted, resuspended in PBS containing 20 mM imidazole, and lysed using a cell disruptor (Constant Systems). After the lysates were clarified by centrifugation (26,600 x g, 1.5 h, 4 °C) the proteins were purified by Ni-NTA chromatography as previously described^41^. His-tagged Skp was obtained from MyBioSource.

### Coupled *in vitro* transcription/translation and OMP folding assays

OMPs were synthesized using the PURExpress coupled transcription-translation system (New England Biolabs) according to the supplier’s instructions, except that reactions were supplemented with 2 μM SurA (or other chaperone) and pre-sonicated 1-palmitoyl-2-oleoyl-glycero-3-phosphocholine (POPC) proteoliposomes containing the Bam complex (0.5 μM). Typical 10 μL reactions (used for single timepoint experiments) also contained murine RNase Inhibitor (8 U) and 0.4 μL FluoroTect Green_Lys_, a lysine-charged tRNA labeled with the fluorophore BODIPY-FL at the ε position (Promega). The latter reagent was used to incorporate fluorescent lysine residues into the protein synthesized *in vitro*. After all of the other reaction components were combined, a pET28b-based plasmid encoding the OMP of interest was added to a final concentration of 10 ng/μL. Unless otherwise noted, transcription/translation reactions were then conducted at 37 °C. After a typical 30 min incubation, reactions were stopped by placing the tubes on ice and adding RNase A (0.5 mg/mL). Proteinase K (30 μg/mL) was then added to some reactions. Protease digestions were conducted for 15 min on ice and halted by the addition of 8 mM PMSF and 2x SDS-PAGE sample buffer.

To analyze the kinetics of OMP assembly, reactions were assembled as described above but volumes were scaled up as necessary (up to 80 μL) and Bam POPC proteoliposomes were omitted. After transcription/translation reactions were initiated (5 min incubation at 37 °C for EspPΔ5 and OmpA, 8 min for OmpG), translation re-initiation was halted by the addition of 10 μM oncocin^42^ (Onc112: VDKPPYLPRPRPPRrIYNr-NH_2_, synthesized and HPLC purified by the Facility for Biotechnology Resources, Center for Biologics Evaluation and Research, FDA). After the initial rounds of translation were allowed to reach completion (typically by incubating reactions at 37 °C for an additional 3 min), Bam POPC proteoliposomes (0.5 μM) were added. For OmpG reactions, this step was omitted and the proteoliposomes were added immediately after the Onc112. At various time points a 3–5 μL sample was removed, added to RNaseA (1 mg/ml), and placed on ice to stop the assembly reaction.

In some experiments OMPs were isolated from inclusion bodies, solubilized in 8 M urea, and added to assembly reactions as previously described^30^.

### Analysis of OMP folding

Aliquots of assembly reactions were mixed with SDS-PAGE sample buffer and either heated at 95 °C for 5 min or left unheated before proteins were resolved on 8–16% NuPAGE minigels (Thermo Fisher Scientific). In general, assembly was assessed by comparing heated and unheated samples and monitoring the appearance of a fast migrating species in the absence of heat that corresponds to the folded form of the OMP. The assembly of EspP∆5 was also analyzed by monitoring the cleavage of the protein (and the appearance of a free β barrel domain fragment) in an autocatalytic reaction that requires the protein to fold into a native conformation. To separate the folded and unfolded forms of OmpG, SDS-PAGE was conducted at 4 ° C. Fluorescently-labeled *in vitro* translated OMPs were visualized using an Amersham Typhoon scanner at an excitation wavelength of 488 nm. The folded fraction was quantitated using ImageJ software and plotted or fit to exponentials using Igor Pro as described^30^. In experiments in which the assembly of urea-denatured OMPs was analyzed, proteins were detected by Western blot as previously described^30^.

## Data availabilty

We declare that all of the data generated during this study are available from the corresponding author upon reasonable request.

## Supplementary information

Supplementary information.
