# Supplementary material for: Bam complex-mediated assembly of bacterial outer membrane proteins synthesized in an in vitro translation system
Source: Sci Rep. 2020 Mar 12;10:4557. doi: 10.1038/s41598-020-61431-2 (PMC7067875; doi:10.1038/s41598-020-61431-2)
Supplement: Supplementary file 1 — Supplementary information. [file 41598_2020_61431_MOESM1_ESM.pdf]

**SUPPLEMENTARY INFORMATION for**

# Bam complex-mediated assembly of bacterial outer membrane proteins synthesized in an in vitro translation system

Sunyia Hussain, Janine H. Peterson and Harris D. Bernstein

Genetics and Biochemistry Branch, National Institute of Diabetes and Digestive and Kidney Diseases, National Institutes of Health, Bethesda, MD 20892-0538 USA

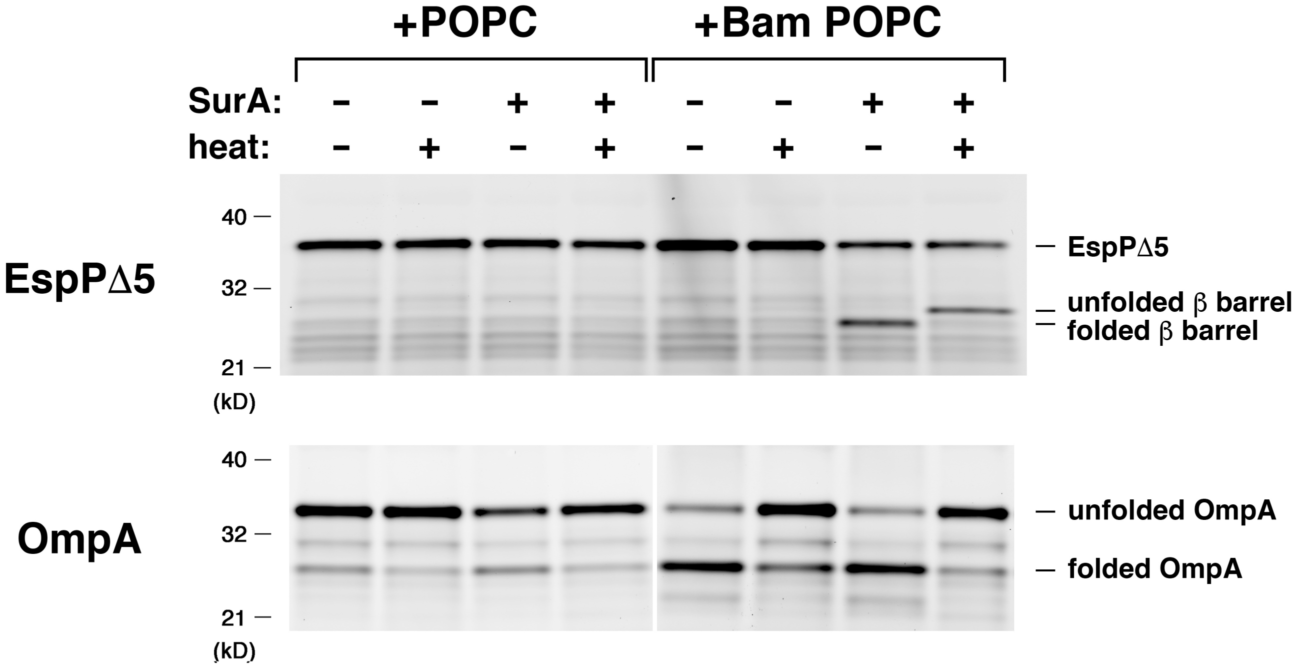


**Figure S1.** OMPs synthesized in vitro are not assembled efficiently in the presence of empty liposomes. PURExpress coupled transcription/translation reactions supplemented with BODIPY-FL-ε-Lys-tRNA$^{Lys}$ were programmed with a plasmid encoding EspPΔ5 or OmpA under the control of a T7 promoter and incubated in the presence or absence of SurA with either POPC liposomes or Bam POPC proteoliposomes at 37° C for 30 min. Aliquots were then removed from each reaction and heated to 95° C or left unheated, and proteins were resolved by SDS-PAGE. The OMPs were detected based on the incorporation of fluorescently tagged lysine residues during synthesis.

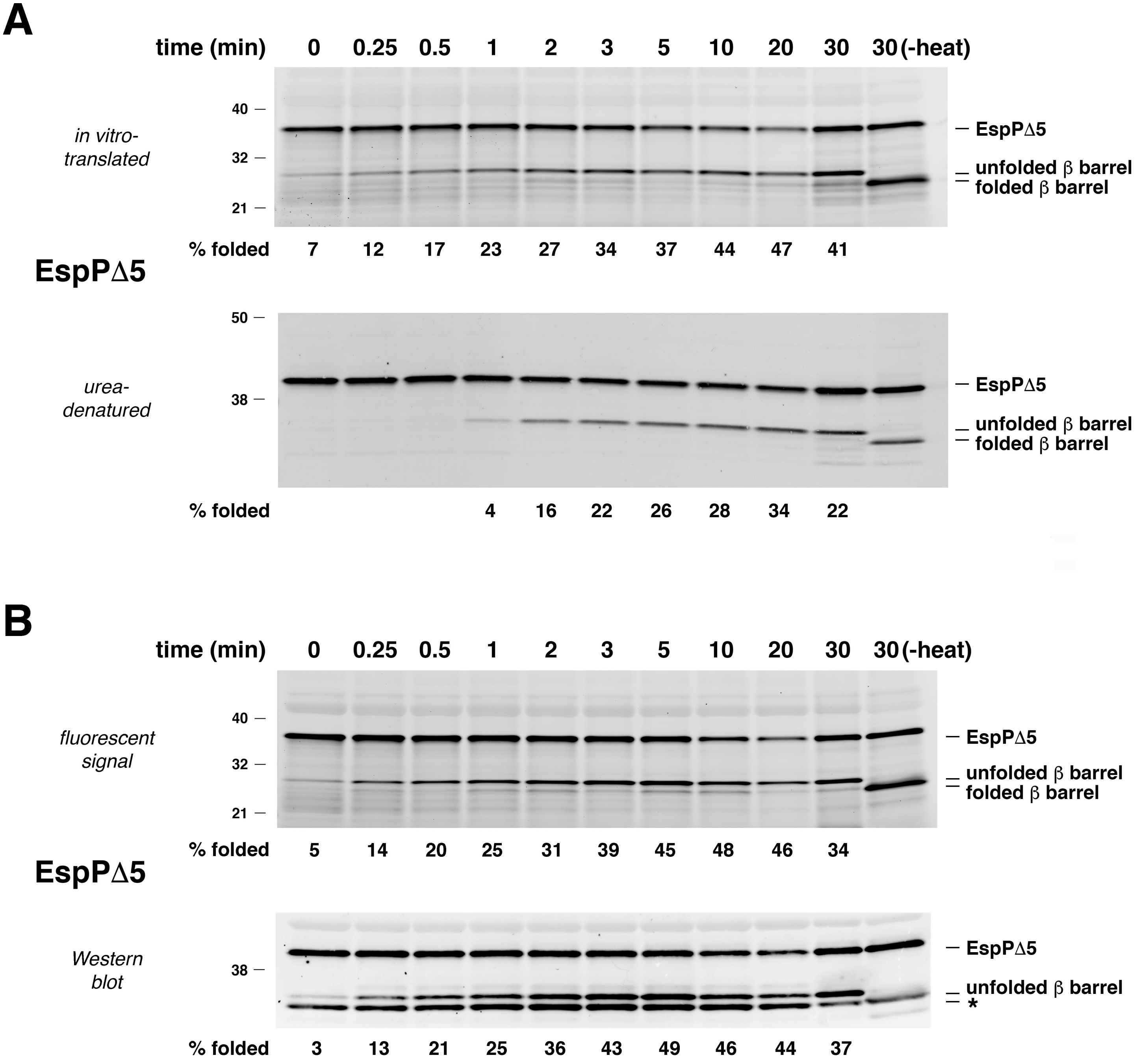


**Figure S2.** Folding kinetics of in vitro translated and urea-denatured EspPΔ5 examined in parallel assays. (A) A PURExpress reaction supplemented with BODIPY-FL-ε-Lys-tRNA$^{Lys}$ and SurA was programmed with a plasmid encoding EspPΔ5 under the control of a T7 promoter and incubated at 37° C for 5 min. Following the addition of Onc112 the reaction was incubated for a further 3 min. Bam POPC proteoliposomes were then added and aliquots were removed at various timepoints. Fluorescently-labeled protein was detected after SDS-PAGE. In parallel, urea-denatured EspPΔ5 was added to an assembly assay that contained SurA and Bam POPC proteoliposomes derived from the same lots. Aliquots were removed at the same time points, and unprocessed EspPΔ5 and the EspPΔ5 β barrel were detected by Western blot following SDS-PAGE using an antiserum generated against an EspP C-terminal peptide. EspPΔ5 assembly was assessed by determining the percent of the protein that underwent self-cleavage. (B) The PURExpress reaction and assembly assay described in part A were repeated and fluorescently-labeled polypeptides were detected after SDS-PAGE (top). The polypeptides were then transferred to nitrocellulose, and unprocessed EspPΔ5 and the EspPΔ5 β barrel were redetected by Western blot using the antiserum described in part A (bottom). An unidentified background band that comigrates with the folded form of the EspPΔ5 β barrel (and that is presumably present in the PURExpress system) is denoted with an asterisk.

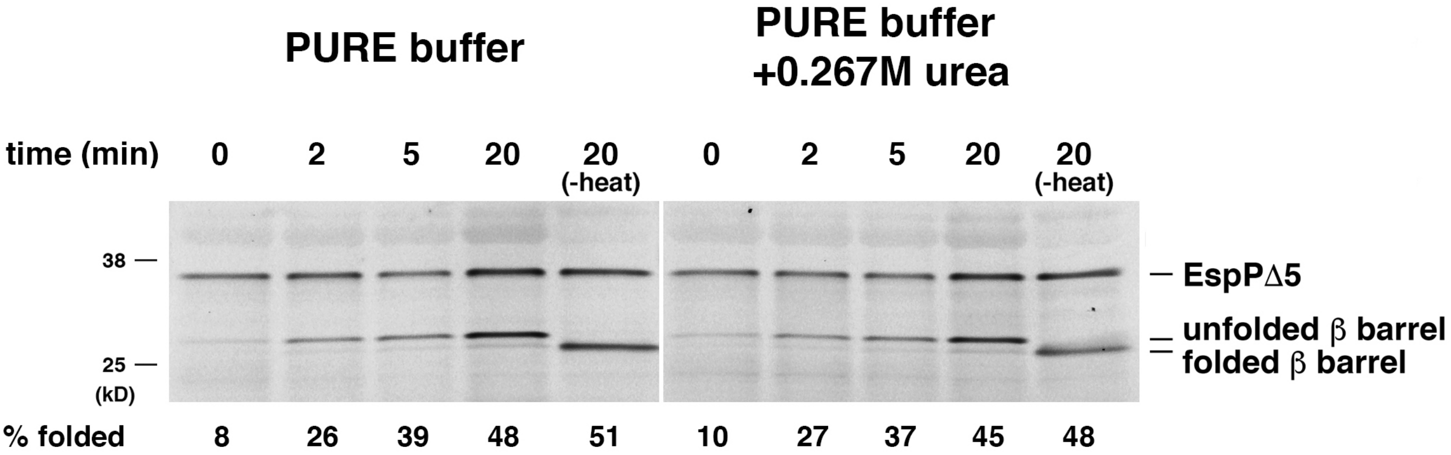


**Figure S3.** The assembly of EspPΔ5 synthesized in vitro is not affected by the addition of urea. Two PURExpress reactions supplemented with BODIPY-FL-ε-Lys-tRNA$^{Lys}$ and SurA were programmed with a plasmid encoding EspPΔ5 under the control of a T7 promoter and incubated at 37° C for 5 min. Following the addition of Onc112 reactions were incubated for a further 3 min. Bam POPC proteoliposomes were then added to the first reaction while both Bam POPC proteoliposomes and urea (final concentration=0.267M) were added to the second reaction. Aliquots were removed at various timepoints, and fluorescently-labeled protein was detected after SDS-PAGE. EspPΔ5 assembly was assessed by determining the percent of the protein that underwent self-cleavage.
